# Chicago Pathological Society

**Published:** 1884-04

**Authors:** J. H. Tebbetts

**Affiliations:** Secretary


The society, on motion, adjourned.

J. H. TEBBETTS, Secretary.

---

### CHICAGO PATHOLOGICAL SOCIETY.

Regular meeting, November, 1883.

The society was called to order by the President, Dr. Angear. The minutes of the last meeting were read and approved.

Dr. F. C. Knight was elected to membership unanimously. Dr. R. J. Curtiss was proposed for membership by Drs. Lyman, Lydston, and Harper.

The society then listened to the reading of an interesting paper by Dr. G. Frank Lydston, entitled, "Some of the Fallacies of Modern Sanitation, with Especial Reference to the Sewage System in Relation to Zymotic Disease."

The subject comprised a variety of topics, and its discussion was not a matter of recent date, but had been under consideration many years, and afforded material worthy of thought to-day. Colleges should instruct graduates in the elements and essentials of sanitary engineering.

The sewage system affords a most potent cause of sickness, and in the large cities the majority of cases of illness come under the name of germ diseases.

The term "modern improvements" he considered sarcastic and incorrect. They served for extension, not prevention, of germ-bearing material. He doubted if there were a single perfect plumber in town. Are water-closets in expensively plumbed residences perfectly trapped and ventilated? The author considered not by any means. The same charge was true, and more especially so, in case of stationary washstands in sleeping rooms. In water-closets, gas always remains beneath the trap, waiting to puff upward when the closet is used and the trap opened. Many varieties of traps were then described, sufficiently to point out the defects of each.

The noxious gases, germ-bearing, may be treated chemically. Solutions placed between double traps answer this purpose. The "germicide," a patented arrangement, was considered good, although imperfect.

In conclusion, water-closets should be detached from the main portion of the dwelling, with a well ventilated passage leading to them.

As concerns stationary washstands, how can the gases be kept below the trap? It was about impossible to do this. The heated air of the room attracts the gases thereto, in direction of the least resistance, and in the sense of smell even there was no protection. Sewer gas was a varying compound, and there was no distinct odor in many cases. Stench was a blessed warning when present.

Cases were cited proving beyond doubt that scarlet fever had been conveyed from house to house, along a street, the germs being inhaled only by those persons sleeping in rooms having direct communication, by means of stationary washstands, with the sewer.

The street mains may be flushed daily. This as done in Paris is valuable.

The ventilating shaft in the house should open into a chimney in constant use, as a warm current is required. No better arrangement could perhaps be devised than the complete detachment of all "modern improvements," in way of plumbing, from the dwelling, and at all events remove all stationary washstands from sleeping rooms. Sewer gas is absorbed by the walls of a house if porous, just as is "crowd poison" in hospitals and barracks, making them untenable, until disinfected by chlorine gas.

The question arises, how are the cities to escape zymotic diseases as nearly as possible? Clean and keep cleaning the alleys, empty the cesspools and allow the sunlight to penetrate them, but use no disinfectant drugs to cover the stenches; remove the cause and disinfectants are needless.

Finally, in the sick room allow a liberal supply of fresh air, thus deodorizing and disinfecting, but dispensing with carbolic acid and such chemicals.

Owing to the lateness of the hour, discussion by the society was postponed.

The society then listened to the reading of a paper by Dr. R. J. Curtiss on a kindred topic: "The Disposal of Chicago Sewage." An abstract of this interesting paper, to be appreciated, should be fully as complete as was the paper itself. At times very amusing, yet truthful in statistics, and accurate in general statement, the paper commanded the closest attention of the society, and on conclusion the author received, on motion of Dr. Lyman, a vote of thanks of the society. Discussion was again postponed on account of the lateness of the hour.

The society then proceeded to the transaction of its miscellaneous business, and first received the report of the committee on place of meeting. Various locations were discussed, but on motion the society granted committee one month longer, advising it possible, the engagement of a suitable parlor in the Washingtonian Home. Dr. C. W. Earle informed the society that he would secure the above room for the next meeting, but was unable to state whether such arrangement could be made a permanent one.

Among those present were Drs. Angear, Brower, Danforth, Dobbin, Earle, Harper, Lyman, Root, Seely, Tagert, Twining, Tebetts and a number of visitors.

